# Origin of Structural Variations in Amorphous SiO_2_ Generated by Melt-Quench Simulations

**DOI:** 10.1021/acs.jpcc.6c00944

**Published:** 2026-05-08

**Authors:** Colton Dechant, Aishwarya Muralidhar, Ying Ma

**Affiliations:** † Department of Materials Science and Biomedical Engineering, 14747 University of Wisconsin-Eau Claire, 105 Garfield Ave., Eau Claire, Wisconsin 54701, United States; ‡ Department of Materials Science and Engineering, 2647 The Ohio State University, 140 W. 19th Avenue, Columbus, Ohio 43210, United States

## Abstract

Melt-quench simulations are widely used to generate structural models of amorphous silicon dioxide (a-SiO_2_), a technologically important material. However, reported results often diverge—particularly regarding the presence of short Si–Si distances near 2.4 Å, typically interpreted as structural defects. Using extensive ab initio molecular dynamics simulations, we demonstrate that these inconsistencies originate from insufficient equilibration of the liquid prior to quenching. We identify two distinct liquid regimes—a high-energy state (HES) and a low-energy state (LES)—and show that quenching from the HES consistently produces defects such as edge-sharing SiO_4_ tetrahedra and 3-fold-coordinated Si atoms, whereas quenching from the LES yields perfectly coordinated, defect-free amorphous structures. The transition between these regimes depends sensitively on temperature and thermostat friction parameters, explaining the variability observed in previous studies. These findings underscore the critical role of liquid equilibration in generating reliable structural models for amorphous materials using the melt-quench method.

## Graphical abstract

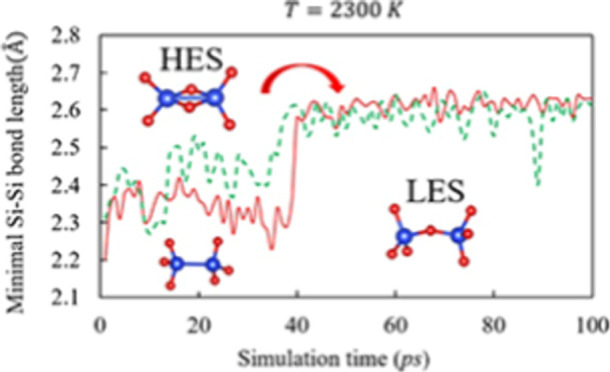

## 1 Introduction

Amorphous materials underpin a wide spectrum of modern technologies, including electronics, catalysis, photovoltaics, energy conversion and storage, and biomedical devices.^1–3^ Achieving precise control over their functional properties requires a detailed understanding of their structural characteristics, particularly at the atomistic scale. However, the absence of long-range order fundamentally limits the structural information accessible through experimental characterization. Techniques such as X-ray and neutron diffraction provide only ensemble-averaged structural signatures and offer little insight into chemical ordering.^3–5^ Computer simulations have therefore become indispensable for probing the atomic-scale structure of amorphous systems. Methods ranging from ab initio and classical molecular dynamics to Monte Carlo simulations and emerging machine-learning-based approaches now enable the generation of detailed structural models that reveal features inaccessible to current experimental techniques.^4–8^

Among amorphous materials, amorphous silicon dioxide (a-SiO_2_) stands as one of the most extensively studied, both experimentally and computationally. Its structure is commonly described as a continuous random network of corner-sharing SiO_4_ tetrahedra, in which short-range order is preserved despite the loss of long-range periodicity.^9^ Yet the intrinsic structural disorder of a-SiO_2_ poses a persistent challenge in experimental characterization: multiple distinct local configurations can reproduce the same experimental observables, preventing unique structural determination.^10^ Computational studies face a similar ambiguity. Different simulations often report inconsistent structural features; for example, some studies report a small peak or shoulder near 2.4 Å in the Si–Si pair distribution function (PDF)^11–13^ —typically attributed to various structural defects^14^—while others do not.^15–18^ This inconsistency may be more prevalent because partial PDFs are not always reported,^14,19,20^ although sometimes the existence of such a peak can be inferred.^14^ Despite such a discrepancy, the simulated total PDFs generally agree with experimental measurements, making it difficult to validate or refute any particular structural model.

Most computational studies generate a-SiO_2_ by rapidly quenching liquid SiO_2_ from high temperature, a natural choice given the structural similarity between the liquid and amorphous phases. Using ab initio molecular dynamics (AIMD) simulations, we demonstrate that the variability among melt-quench-generated structures arises primarily from differences in the simulation history. AIMD was chosen over classical MD to minimize possible variations due to the varying quality of empirical force fields, although it should be noted that the inconsistency in the Si–Si peak has been observed in both AIMD^11^ and classical MD simulations.^12^ We show that the defects are not an intrinsic feature but rather a computational artifact originating from insufficient equilibration of the liquid phase prior to quenching, highlighting the importance of rigorous validation of computationally generated amorphous structures.

## 2 Computational Methods

We began by generating a stoichiometric random mixture of Si and O atoms comprising 120 atoms in total at the experimental density of 2.2 g/cm^3^, which was then equilibrated for 100 ps at high temperature under the constant volume and constant temperature (*NVT*) ensemble. Three independently generated random mixtures, referred to as system I, II, and III, respectively, were prepared to enhance the statistical reliability of the results. Melting temperatures, referred to as the temperature at which the melting simulation is performed, that range from 1900 K to 3100 K were tested to explore the effect of temperatures on the resulting amorphous structure. A Langevin thermostat^21^ was used to maintain the temperature of the system, and two different friction coefficients, γ = 10 ps^–1^ and γ = 50 ps^–1^, were used and compared. We note that production runs in AIMD simulations are typically less than 30 ps due to their high computational cost. Here, 100 ps was used to promote the formation of a homogeneous mixture. As will be shown later, even 100 ps equilibration may not be sufficient depending on the choice of simulation parameters. The melt was then quenched to 300 K in 5 ps under the *NVT* ensemble, followed by equilibration in the constant pressure, constant temperature (*NPT*) ensemble at 300 K for 30 ps. The Parinello–Rahman algorithm was used to control the pressure of the system.^22^

All calculations were performed using the Vienna Ab initio simulation package (VASP),^23,24^ employing the PBE functional^25^ and the projector-augmented wave method.^26^ Brillouin zone sampling was performed using the Γ point only. A time step of 1 fs was used for all calculations.

## 3 Results and Discussion

Because inconsistencies in the literature center on a short Si–Si bond length at about 2.4 Å, we monitored the minimal Si–Si bond length during the 100 ps melting simulation. Figure 1 plots the variation of the minimal Si–Si bond length for system I at four melting temperatures, 1900 K, 2300 K, 2700 K, and 3100 K. At 1900 K, the minimal Si–Si bond length fluctuates near 2.3 Å (Figure 1a), consistent with thermal vibrations. At 2300 K, a structural transition occurs at about 40 ps, after which the minimal Si–Si bond length increases to about 2.6 Å, as shown in Figure 1b. We calculated the energies of five configurations each before and after the transition, and an energy lowering of 0.11 ± 0.01 eV/atom accompanying the transition was observed. We denote the structure before the transition as the high-energy state (HES) and after the transition as the low-energy state (LES). Given that the thermal energy (*kT*) at 2300 K is around 0.20 eV and is comparable to the free energy difference between HES and LES, thermal fluctuations can promote transitions between the two states, leading to some populations of the HES even after the transition to the LES. This is evident at higher temperatures (Figure 1c,d) for γ = 10 ps^–1^, where the system intermittently samples both states. The thermostat friction coefficient also affects the structure of the melt. At 2700 K and γ = 10 ps^–1^, the system remains mostly in the LES except between about 20 ps–40 ps, whereas for γ = 50 ps^–1^ the minimal Si–Si bond length fluctuates near 2.3 Å and no transition to the LES is observed.

**Figure 1:**
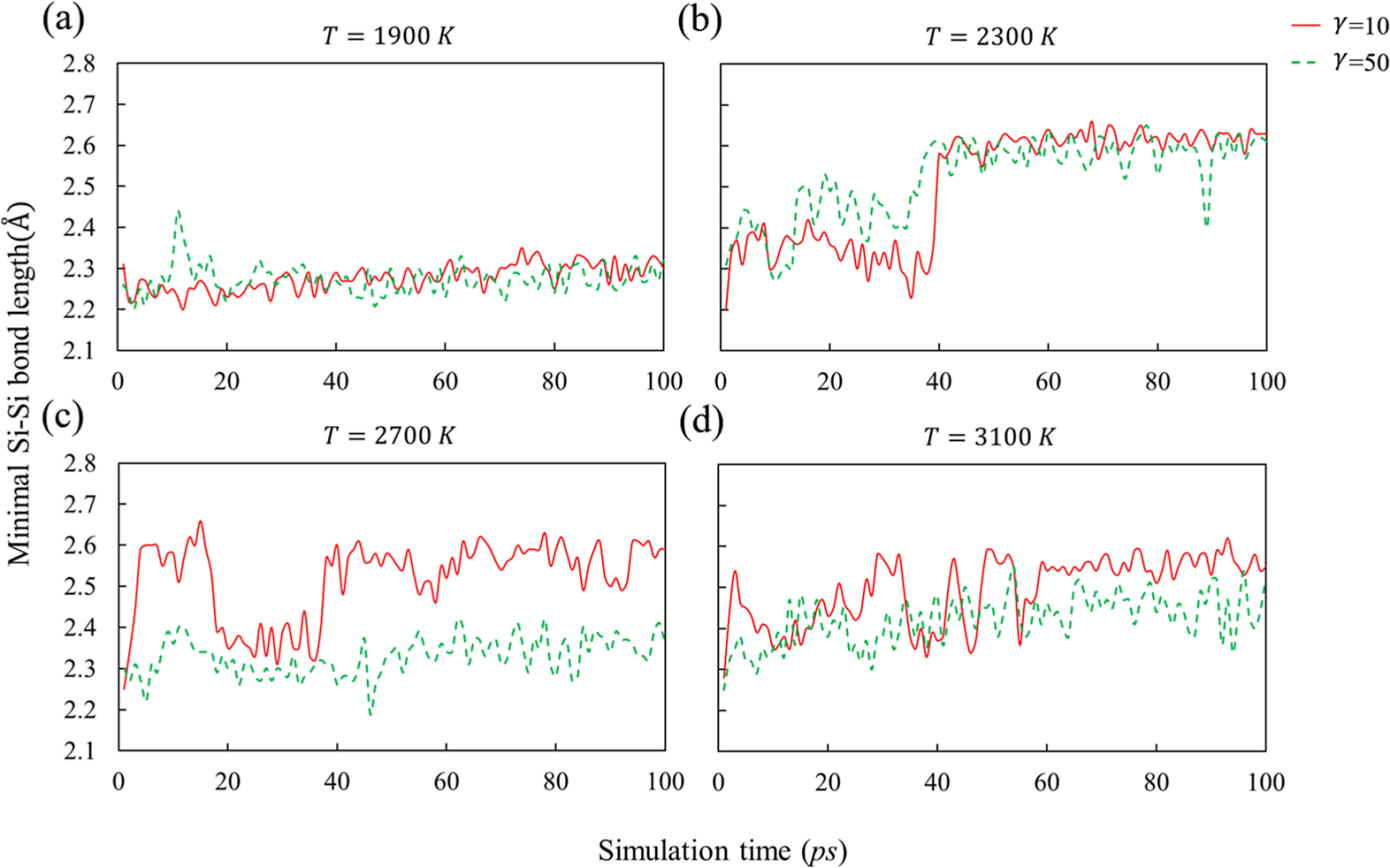
Variation of the minimal Si–Si bond length during melting at different temperatures for system I. Solid line corresponds to a Langevin thermostat friction coefficient of 10 ps^–1^, while the dashed line corresponds to a friction coefficient of 50 ps^–1^.

The variation of the minimum Si–Si bond length in systems II and III resembles that observed in system I, and the corresponding data are provided in the Supporting Information (Figures S1 and S2, respectively). In all three systems, the transition from the HES to the LES occurs at temperatures of 2300 K or higher. However, no systematic trend in the simulation parameters—such as melting temperature, thermostat friction coefficient, or simulation duration—can be identified, consistent with the use of randomly generated initial structures and the stochastic nature of the Langevin thermostat. Nevertheless, the existence of a structural transition in the liquid state is evident.

To investigate the impact of the liquid-state structure on the resulting amorphous structure, five configurations from the LES (three from 2300 K and two from 2700 K) and five from the HES (three from 2700 K and two from 2300 K) were randomly selected, quenched, and subsequently equilibrated at 300 K. The resulting amorphous structures were then analyzed. All structures quenched from the HES exhibited a small Si–Si peak at about 2.4 Å, whereas no such peak was observed for any structure quenched from the LES. Because all HES-quenched structures are similar to one another, and all LES-quenched structures are likewise similar, a representative Si–Si PDF from each set is selected and shown in Figure 2a. Further analysis shows that the small Si–Si peak can be attributed to two pairs of Si atoms whose bond length fluctuates between 2.3 Å and 2.5 Å. Due to their relatively low concentration, such structural features, while distinct in the Si–Si PDF, are essentially buried in the total PDF as shown in Figure 2b; indeed, the total PDFs for HES- and LES-quenched systems are almost indistinguishable. The Si–O and O–O PDFs of HES- and LES-quenched systems are also very similar, as shown in Figure S3.

**Figure 2:**
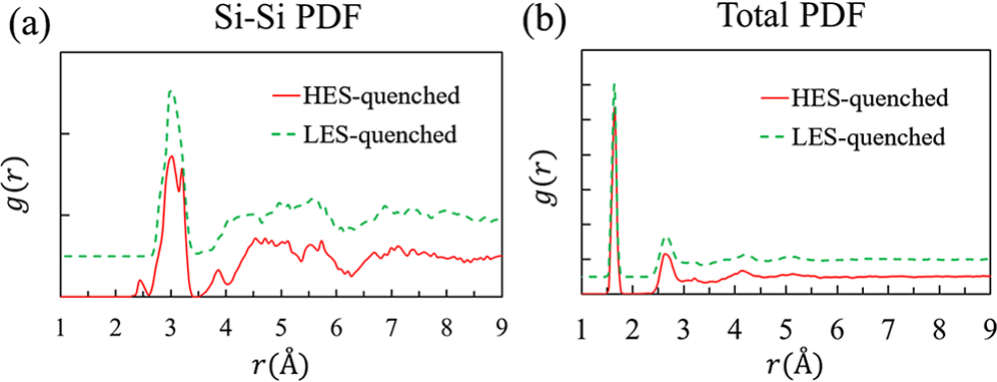
(a) Si–Si pair distribution functions for one representative structure quenched from HES and one from LES. (b) The total pair distribution functions for the same two structures.

As illustrated in Figure 3, the two pairs of Si atoms with a short bond length correspond to two types of structural defects: edge-sharing SiO_4_ tetrahedra (Figure 3a) and under-coordinated Si atoms with three oxygen neighbors (Figure 3b). We note that both types of defects have been previously reported.^11,14^ In particular, the edge-sharing tetrahedra correspond to a two-membered ring structure, and analysis of the ring-size distribution confirms the presence of such defects in the HES-quenched structures. As shown in Figure S4a, the HES-quenched structure exhibits a nonzero population of two-membered rings. Within the two-membered ring structure, the Si–O–Si bond angles are approximately 90°–115°, while the O–Si–O bond angles are 75°–95°.^27,28^ The bond angle distribution of the HES-quenched systems (Figure 4) confirms the presence of small peaks within these ranges, in addition to the dominant peaks at approximately 109° (O–Si–O) and 132° (Si–O–Si). Furthermore, the O–O bond length in a two-member ring is in the range of ∼2.1–2.3 Å,^27,28^ which gives rise to a subtle shoulder in the O–O PDF of the HES-quenched structure (Figure S3b).

**Figure 3:**
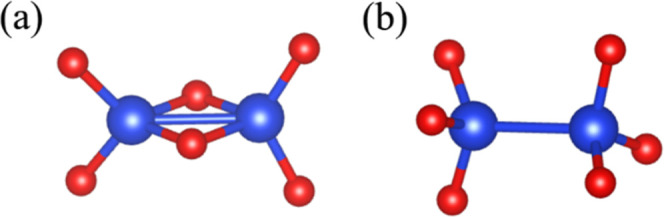
Two types of structural defects observed in HES-quenched samples: (a) edge-sharing SiO_4_ tetrahedra, where each Si atom is coordinated by four oxygen atoms; and (b) under-coordinated Si atoms with three oxygen neighbors. In both cases, the Si–Si bond length is about 2.4 Å. Blue and red spheres represent Si and O atoms, respectively.

**Figure 4:**
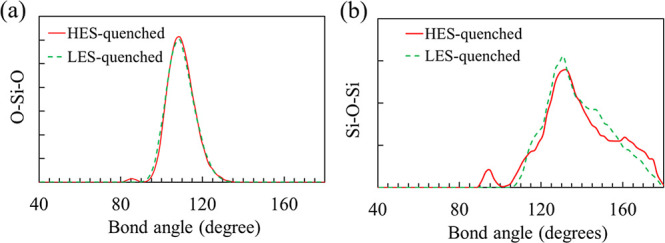
(a) O–Si–O bond angle distribution for one representative structure quenched from HES and one from LES. (b) The Si–O–Si bond angle distribution for the same two structures.

In contrast, both types of defects shown in Figure 3 are absent in all structures quenched from the LES. In LES-quenched systems, there is no Si–Si peak at 2.4 Å (Figure 2a), the two-membered rings disappear with a significant increase in the intensity of six-membered rings (Figure S4a), and only the dominant peaks exist in O–Si–O and Si–O–Si angular distributions (Figure 4). In other words, when the liquid structure is well-equilibrated and has reached the LES during melting, the resulting amorphous structure at 300 K is free of these defects. In fact, all Si atoms in the five LES-quenched amorphous structures are perfectly 4-fold coordinated, and key structural metrics, as summarized in Table 1, agree well with experimental data.

**Table 1:** Structural Characteristics of a-SiO_2_ for a Representative LES-Quenched System^a^

structural data	this work	experimental^29,30^
Si–O bond length	1.63	1.61
Si–Si bond length	3.03	3.08
O–O bond length	2.67	2.63
average Si–O–Si bond angle	138.5	140–150
average O–Si–O bond angle	109.4	109.4–109.7

a Minimal variations are observed among all LES-quenched systems.

All structural differences observed between HES- and LES- quenched systems originate from differences in the liquid structure prior to quenching. As a representative example, we examined system I melted at 2300 K with γ = 10, which was selected because it exhibits a well-defined transition and the LES remains stable following the transition. The ring size distribution, Si–Si and total PDFs, Si–O and O–O PDFs, and bond angle distributions for the HES and LES are shown in Figures S4b and S5–S7, respectively. Characteristic features of the HES, including the presence of two-membered rings, a small peak at 2.4 A in Si–Si PDF, and a peak near 90° in the Si–O–Si bond angle distribution, are absent in the LES. However, the distinctions between the HES and LES at this temperature are significantly less pronounced than those observed in systems quenched to 300 K, owing to increased thermal fluctuations and the resulting smoothing of structural features. Subtle differences, such as a weak shoulder in the O–O PDF and a minor peak near 85° in the O–Si–O bond angle distribution, are no longer discernible. As a result, the existence of the HES to LES transition can hardly be inferred from these commonly used structural metrics. Similarly, the coordination number, another frequently used structural descriptor for a-SiO_2_, fails to give a clear indication of the transition: as shown in Figure S8, a slight increase in the average coordination number of both Si and O atoms is observed at around 40 ps, but the magnitude of this increase is comparable to that of thermal fluctuations. Only the minimal Si–Si bond length (e.g., Figure 1b) provides unambiguous evidence of the transition, although it is not typically monitored in MD simulations. Consequently, structural variations in a-SiO_2_ have not previously been associated with the underlying liquid state structural transition.

A number of classical MD studies^12,31,32^ have examined the effects of cooling rate on the resulting structure of a-SiO2. Structural defects similar to those observed in this study, which manifest as small peaks in the Si–Si PDF and Si–O–Si bond angle distribution, have been reported at high cooling rates and attributed to rapid quenching.^12^ In the present study, the cooling rates range from $3.2\times 10^{14}\frac{K}{s}$ (quenched from 1900 K) to $5.6\times 10^{14}\frac{K}{s}$ (quenched from 3100 K), which are comparable to the highest cooling rates in those studies and are within the range typically employed in AIMD simulations. Notably, even at these extreme cooling rates, no structural defects are observed when the system is quenched from the LES. This observation indicates that the formation of structural defects in a-SiO_2_ is not an intrinsic consequence of rapid cooling, but rather a computational artifact that originates from quenching the liquid while it is in the HES, independent of the cooling rate used. Consistent with this interpretation, such defects disappear upon slower cooling rates, which can be attributed to the system having sufficient time to undergo the HES to LES transition.

It is worth noting that the transition to the LES is not guaranteed. As shown in Figure 1 (as well as Figures S1 and S2), the transition may or may not occur (e.g., no transition was observed at 1900 K even after 100 ps simulation), depending on the choice of simulation parameters such as temperature and thermostat friction coefficient. Conversely, suitable parameters can promote a rapid transition to the LES, thereby reducing the computational cost associated with melting. This is seen in Figure 1c, where the LES first appears in less than 10 ps. Unfortunately, such parameters are not known a priori and depend on the initial structures, which are often randomly generated (Figures S1 and S2). Further complicating the issue is the high temperature used in melting simulations, where thermal fluctuations can lead to intermittent sampling of both HES and LES configurations. Together, these factors explain the inconsistencies in reported structures of a-SiO_2_ and underscore the importance of rigorous validation of computationally generated structural models for amorphous materials.

## 4 Conclusions

In this work, AIMD simulations were performed to evaluate the structure of melt-quench generated a-SiO_2_. A transition from HES to LES was observed during melting; however, the presence of this transition depends sensitively on the choice of computational parameters. As a result, the amorphous structures obtained at 300 K exhibit notable variability, exemplified by inconsistencies in the existence of a small peak near 2.4 Å in the Si–Si PDF and small peaks in Si–O–Si and O–Si–O bond angle distribution. Although only a-SiO_2_ was examined in the present study, similar structural transitions during melting may also occur in other materials. Based on our findings, we recommend conducting melting simulations at temperatures moderately above the melting point of the crystalline phase—high enough to ensure complete melting but not so high as to introduce excessive thermal fluctuations. During this process, the liquid structure should be carefully monitored to detect any potential structural transitions.

While our results suggest that a stoichiometric a-SiO_2_ quenched from a well-equilibrated liquid within the LES contains no short Si–Si bond defects, various defects may still exist in systems that deviate from ideal stoichiometry, for example, those exhibiting oxygen deficiency. Furthermore, undercoordination is inevitable for surfaces and nanoparticles, which may result in structural defects similar to those depicted in Figure 3. For example, classical MD simulations revealed edge-sharing tetrahedra (Figure 3a) on amorphous silica surface.^27,28^ In addition, a small peak in the Si–Si PDF analogous to Figure 2a has been reported for computationally generated nano^33^ and surface amorphous silica.^34^

In melt-quench simulations to generate amorphous structures, the *NPT* ensemble is commonly employed during low-temperature equilibration to determine the density of the amorphous solid. Both *NPT* ^12,14,31^ and *NVT* ^11,13,18,20,35,36^ ensembles are widely used in melting simulations at higher temperatures to obtain a homogeneous liquid. In the present study, the initial structures were randomly generated, and the presence of unphysical bonds may lead to unphysical cell shape and volume variations if the *NPT* ensemble is used. Moreover, *NPT* simulations typically require substantially higher plane-wave energy cutoffs to achieve reliable stress convergence, resulting in a significant increase in computation cost. For these reasons, the *NVT* ensemble was used for melting simulations, while the *NPT* ensemble was employed for equilibration runs at 300 K. This approach is consistent with those adopted in previous studies.^15,17^ Nevertheless, a systematic investigation of how volume fluctuations during melting influence the HES-LES transition, and thus the resulting defect structure, would be of considerable interest, which is left as the subject of a future study.

## Supplementary Material


