# Supplementary material for: Origin of Structural Variations in Amorphous SiO2 Generated by Melt-Quench Simulations
Source: J Phys Chem C Nanomater Interfaces. 2026 May 8;130(20):7148–52. doi: 10.1021/acs.jpcc.6c00944 (PMC13200183; doi:10.1021/acs.jpcc.6c00944)
Supplement: Supplementary file 1 [file jp6c00944_si_001.pdf]

Origin of Structural Variations in Amorphous SiO_2 Generated by Melt-Quench Simulations

Colton Dechant[1], Aishwarya Muralidhar[2], and Ying Ma[1]*

1. Department of Materials Science and Biomedical Engineering, University of Wisconsin-Eau Claire, 105 Garfield Ave., Eau Claire, Wisconsin 54701, United States.

2. Department of Materials Science and Engineering, The Ohio State University, 140 W. 19th Avenue, Columbus, Ohio 43210, United States

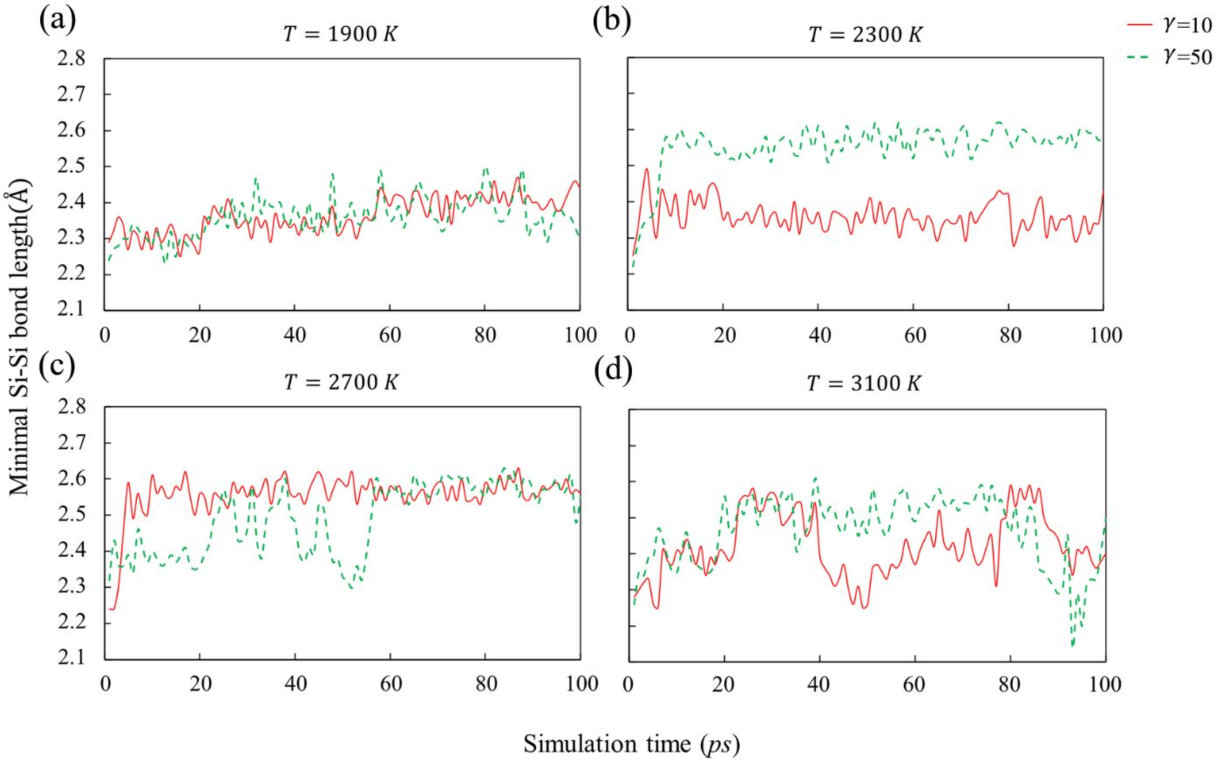

Figure S1. Variation of the minimal Si-Si bond length during melting at different temperatures for system II. Solid line corresponds to a Langevin thermostat friction coefficient of $10\ ps^{-1}$, while the dashed line corresponds to a friction coefficient of $50\ ps^{-1}$.

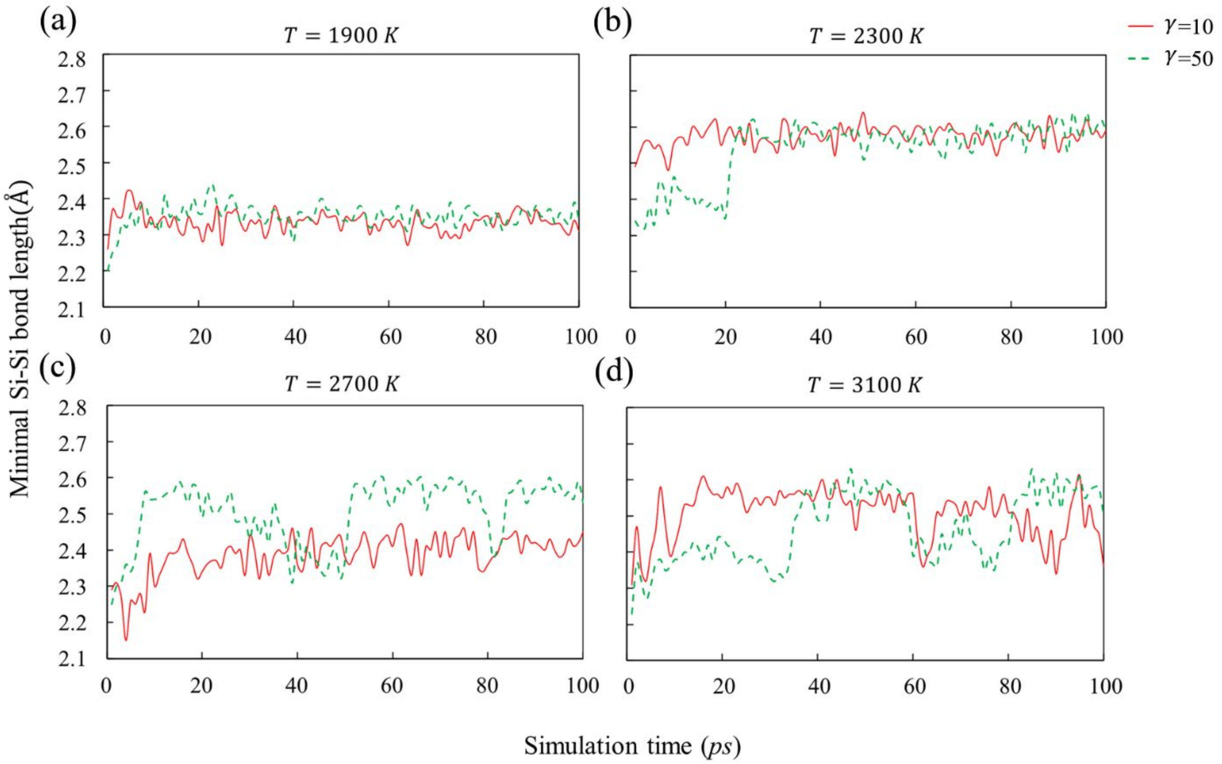

Figure S2. Variation of the minimal Si-Si bond length during melting at different temperatures for system III. Solid line corresponds to a Langevin thermostat friction coefficient of $10\ ps^{-1}$, while the dashed line corresponds to a friction coefficient of $50\ ps^{-1}$.

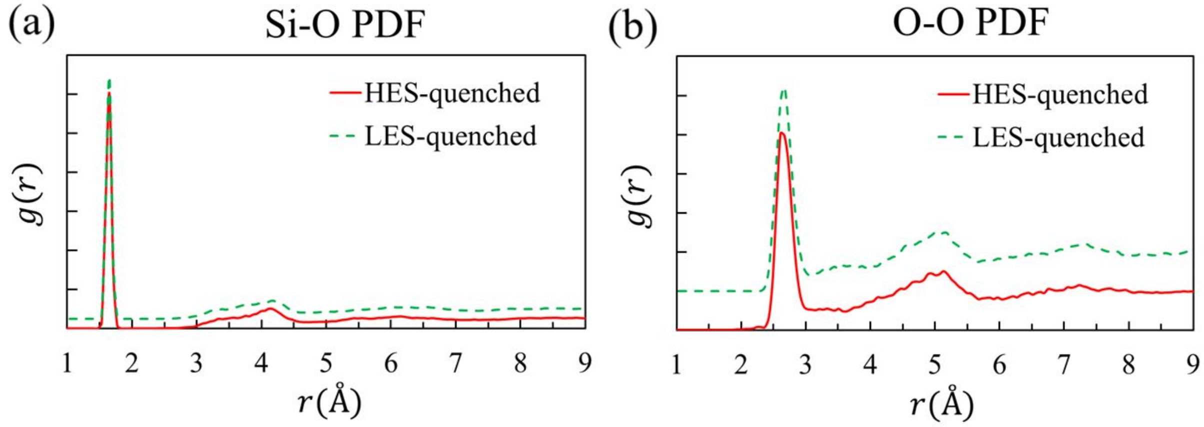

Figure S3. (a) Si-O pair distribution functions for one representative structure quenched from HES and one from LES. (b) O-O pair distribution functions for the same two structures.

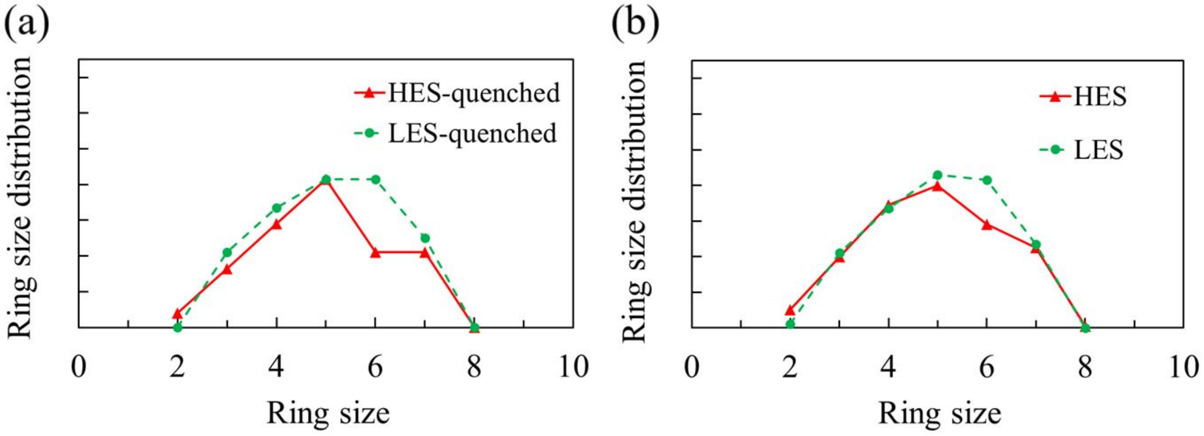

Figure S4. (a) The ring size distribution for one representative structure quenched from HES and one from LES. (b) The ring size distribution before quenching for system I at 2300 K. The distribution was calculated before the transition, which is the HES state, and after the transition, which is the LES state.

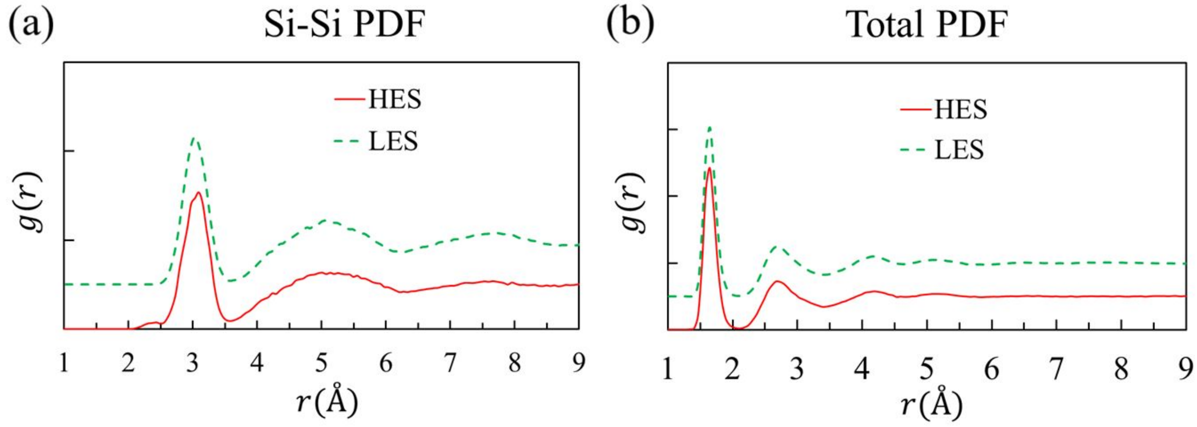

Figure S5. (a) Si-Si pair distribution function for system I at 2300 K in the HES and the LES. (b) The total pair distribution function for system I at 2300K in the HES and LES.

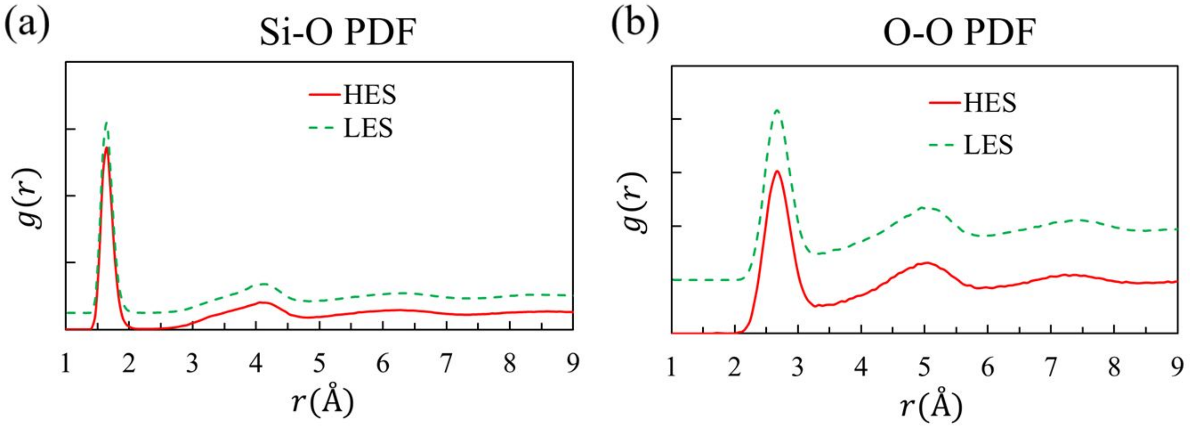

Figure S6. (a) Si-O pair distribution function for system I at 2300 K in the HES and the LES. (b) O-O pair distribution function for system I at 2300K in the HES and LES.

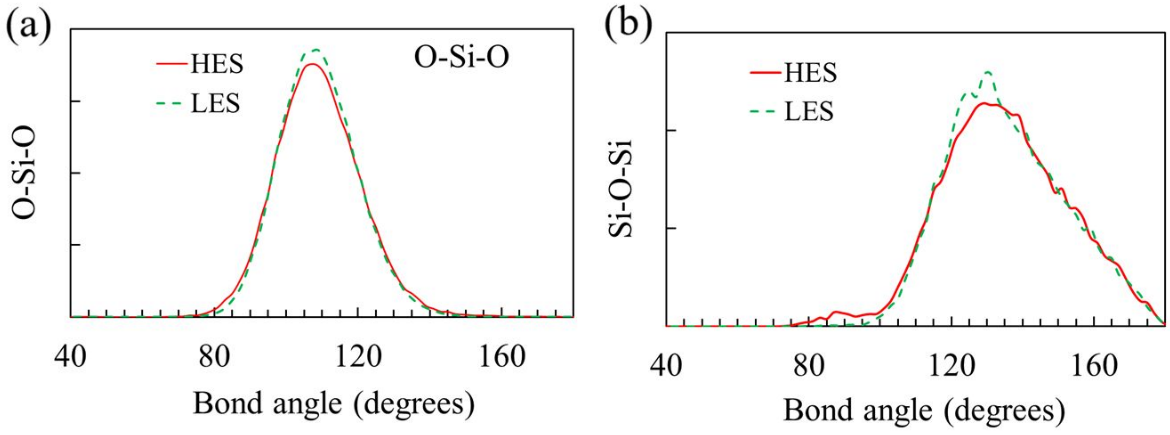

Figure S7. (a) O-Si-O bond angle distribution for system I at 2300 K in the HES and the LES. (b) Si-O-Si bond angle distribution for system I at 2300K in the HES and LES.

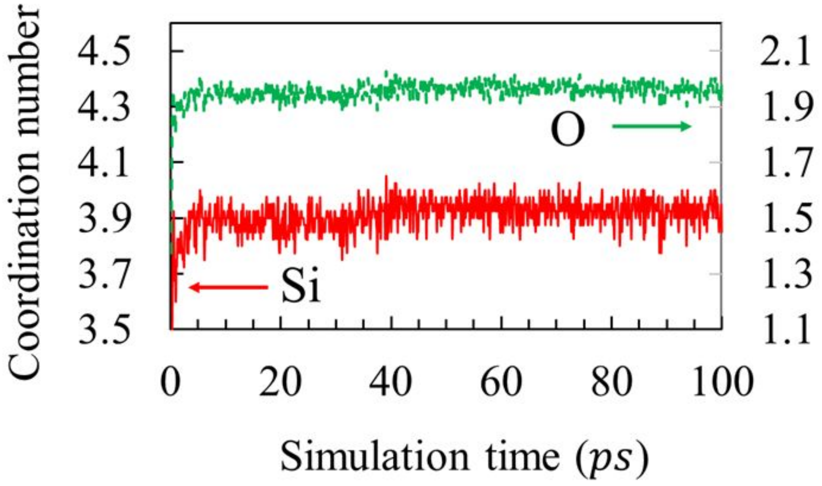

Figure S8. Variation of the Si and O coordination number for system I at 2300 K. A slight increase in the Si and O coordination number is observed at around 40 ps.
